# Comparison of associations suggests mainly distinct pools of genetic risk factors contribute to cisplatin-induced hearing loss and hearing difficulty in the general population

**DOI:** 10.3389/fphar.2025.1577072

**Published:** 2025-08-26

**Authors:** Mohammad Shahbazi, Heather E. Wheeler, Xindi Zhang, Robert D. Frisina, Lois B. Travis, M. Eileen Dolan

**Affiliations:** ^1^Department of Medicine, University of Chicago, Chicago, IL, United States; ^2^Department of Biology, Loyola University Chicago, Chicago, IL, United States; ^3^Departments of Medical Engineering and Communication Sciences and Disorders, Global Center for Hearing and Speech Research, University of South Florida, Tampa, FL, United States; ^4^Department of Medical Oncology, Indiana University, Indianapolis, IN, United States

**Keywords:** cisplatin, cancer, hearing loss, ototoxicity, genetic risk

## Abstract

Cisplatin is an effective chemotherapeutic agent for treating many cancers. However, a major complication associated with cisplatin treatment is ototoxicity. Since the early 2000s, several genetic risk factors linked to cisplatin ototoxicity have been reported. However, the extent to which these genetic risk factors might be shared with those contributing to hearing difficulty in the general population remains unknown. In this study, we investigate if variants with reported links to increased risk of ototoxicity in cisplatin-treated cancer cohorts were also associated with hearing impairment in the general population in the results from a recent meta-analysis (Meta-study; 501,825 participants). Importantly, no significant associations were identified. We also compared association results from our recent genome-wide association study (GWAS) for hearing loss in male testicular cancer survivors (Pt-study; 1,071 participants) with those from both Meta-study and a meta-analysis of the male subset (Male-study; 223,081 participants). We observed evidence for colocalization at the rs7952909 locus across the Male-study and Pt-study results, however, with opposite directions of effects. Across pairwise comparisons, only two variants with matching directions of effects reached significance when relaxed selection cutoffs (10^−3^ or 10^−4^) were used. Collectively, our results suggest that genetic risk factors for cisplatin-induced ototoxicity and those for hearing difficulty in the general population are largely distinct.

## Introduction

Hearing impairment is estimated to be the second most common impairment globally, affecting more than 10% of the population (Vos T. et al., 2016). Its prevalence among US adults is 14.1% within the speech frequency range (18.6% in Males; 9.6% in Females) and 31% at higher frequencies (42% in males; 20% in females), with hearing impairment about twice as common in the male population compared to females (Hoffman et al., 2017). A diverse array of risk factors have been associated with hearing impairment including age, sex, race, hereditary syndromes, congenital infections, hypoxia or low weight at birth, viral and middle ear infections, meningitis, hyperbilirubinemia, hypertension, diabetes, otosclerosis, impacted ear wax, head or ear trauma, loud noise exposure, nutrient deficiencies and, exposure or use of ototoxic agents and, smoking (Hoffman et al., 2017; WHO Team: Sensory Functions, 2021). Hearing impairment has been identified as a risk factor for cognitive decline and dementia (Loughrey et al., 2018).

In recent years, several studies have leveraged the power of large datasets, including the UK Biobank, to identify genetic risk factors associated with hearing problems in the general population (Wells et al., 2019; Ivarsdottir et al., 2021; Trpchevska et al., 2022; De Angelis et al., 2023). Identification of genetic risk factors involves evaluating the relationship of DNA genetic variants, such as single nucleotide polymorphisms (SNPs), with a phenotype (i.e., hearing problems). Genome-wide association studies (GWAS) simultaneously test associations of millions of variants across the genome. The identification of associated genetic variants provides an impetus for studying the nearby genetic regions for functional elements (e.g., genes) to establish their potential mechanisms of action.

Platinum compounds, including cisplatin, are widely used anticancer drugs (Rottenberg et al., 2021); however, ototoxicity is one of the primary complications associated with cisplatin use (McHaney et al., 1983; McKeage, 1995). Similar to hearing problems in the general population, several studies have reported associations of genetic risk factors for cisplatin ototoxicity (Peters et al., 2000; Oldenburg et al., 2007; Riedemann et al., 2008; Caronia et al., 2009; Ross et al., 2009; Xu et al., 2012; 2015; Choeyprasert et al., 2013; Pussegoda et al., 2013; Yang et al., 2013; Brown et al., 2015; Lanvers-Kaminsky et al., 2015; Olgun et al., 2016; Talach et al., 2016; Vos H. et al., 2016; Drögemöller et al., 2017; Lopes-Aguiar et al., 2017; Spracklen et al., 2017; Drogemoller et al., 2018; Lui et al., 2018; Tserga et al., 2019; Langer et al., 2020; Meijer et al., 2021; Garcia et al., 2023; He et al., 2023; Hurkmans et al., 2023). Our group has recently examined the well-characterized Platinum Study cohort to identify the ototoxic effects of cisplatin in testicular cancer survivors (Zhang et al., 2022).

To our knowledge, the extent of overlap among common genetic risk factors associated with cisplatin-mediated ototoxicity and hearing impairment in the general population has not been previously evaluated. A high overlap in genetic risk factors could be an indicator of shared genetic architecture for hearing loss, including similarity in the underlying cellular and molecular pathways. This could serve as the basis to formulate broad-spectrum predictive genetic models for hearing impairment and raise the prospect of developing widely applicable therapeutic and preventive measures for hearing deficits. In contrast, if a limited pool of common genetic risk factors is identified, this would support the development of environmental risk factor-specific genetic models, prevention, and management strategies.

In this study, we first examined variants with reported relationships for cisplatin ototoxicity for their associations with hearing difficulty in the general population using a recent meta-analysis (Meta-study) (De Angelis et al., 2023). Next, we used this meta-analysis and our recent GWAS for cisplatin ototoxicity in a pairwise comparison to explore the extent to which shared genetic risk factors exist across the two studies. Since our Pt-study employs a cohort of male participants, we extended our pairwise comparison to the meta-analysis of hearing impairment in the male subset (Male-study) (De Angelis et al., 2023). Collectively, results from these comparisons are expected to indicate the extent to which shared genetic risk factors may exist across cisplatin-induced ototoxicity and hearing impairment in the general population.

## Methods

### Preparation of summary statistics from genome-wide association studies

Summary statistics from meta-analyses of genetic associations of hearing difficulty in the general population (Meta-study, incorporating UK Biobank, the Nurses’ Health studies (I and II) and the Health Professionals Follow-up Study cohorts), in the male subset (Male-study, incorporating UK Biobank and the Health Professionals Follow-up Study cohorts) and, hearing loss in testicular cancer survivors (Pt-study; all males) were obtained from their respective studies (De Angelis et al., 2023; Zhang et al., 2022). In these studies, population stratifications were controlled for by the inclusion of the top ten genetic principal components as covariates during genome-wide association analyses. The genotypes from the 1000 genomes (1000 Genomes Project Consortium et al., 2015) (2016-05–05 primary release, build 37, 2,504 samples, without singletons, with KING-based pedigree corrections and, with information annotations, https://www.cog-genomics.org/plink/2.0/resources) were filtered to include samples with European ancestry and to include autosomal variants. Variant identifiers, consisting of chromosome, position, and allele information were used to remove duplicates and for harmonization across datasets. PLINK2, V2.00a6LM (Chang et al., 2015) (https://www.cog-genomics.org/plink/2.0) with the 1000 genomes as the reference panel was used for clumping of harmonized variants (*r* ^2^ threshold of 0.1 and distance of 500 kb) (Supplementary Figure S1) or to calculate linkage disequilibrium (LD) estimates for variants in proximity to missing variants in Meta-study. Nomenclature for reporting of SNPs included standard identifiers referred to as reference SNP-cluster IDs (rs IDs), which include a “rs” prefix followed by a unique number (Sherry et al., 1999).

### Associations with hearing difficulty in the general population for variants with previously reported associations with hearing loss in cancer cohorts

We performed a literature search to identify genetic variants associated with hearing loss in cisplatin-treated cancer survivor cohorts (Table 1). For genome-wide association studies in cancer cohorts, based on the data availability, variants below suggestive association thresholds 10^−5^ (Meijer et al., 2021; Garcia et al., 2023; He et al., 2023; Hurkmans et al., 2023) or 5 × 10^−6^ (Xu et al., 2015) were included. LDPair webtool (Machiela and Chanock, 2015) (https://ldlink.nih.gov/) with European ancestry was then used to identify variant pairs in LD (*r* ^2^ > 0.1) within 500 kb. If the variant pair were from the same study, the variant with the more significant association was maintained. In a case where a pair of variants in LD were from different studies, both variants were included in the comparisons but were considered as one variant in the calculation of the Bonferroni threshold of proxy variants. When variants from the cancer cohorts were absent in Meta-study association results, we checked within 500 kb of the missing variant for the presence of a proxy variant that would pass a Bonferroni-corrected threshold for significance. If any significant proxy variants were identified, their LD with the missing variant was estimated using the 1000 genomes reference panel (Supplementary Table S1).

**TABLE 1:** List of variants with previously reported associations with hearing loss in cancer survivors tested for their associations in Meta-study.

Var	Study	Chr:Pos:A1:A2	Var	Study	Chr:Pos:A1:A2	Var	Study	Chr:Pos:A1:A2
1	A	1:11028025:CTC:CTC​CTC^1*^	31	B	5:12641583:A:G^1^	61	H	12:125980301:T:C
2	B	1:70148282:T:C	32	B	5:31960647:T:G	62	C	12:126635113:A:G
3	C	1:112133910:T:C	33	D	5:77349787:C:T^1^	63	H	13:20749239:T:C
4	D	1:161380911:A:G	34	B	5:78578496:T:G^1^	64	N	13:27399338:A:G
5	D	2:655222:A:G	35	D	5:79870334:A:C	65	C	13:75672116:T:C
6	B	2:8811354:A:C	36	B	5:174470036:G:A^4^	66	D	13:76274802:A:G
7	C	2:21648283:T:C	37	B	6:8298796:G:A^1^	67	B	13:110743126:T:C^2^
8	E; D; F; G	2:54395259:A:G	38	O; P	6:18139802:A:T	68	B	14:20947158:A:G^3^
9	D	2:56139436:A:G^1^	39	H	6:28274651:A:G	69	C	14:22028781:A:G
10	D	2:118189068:T:C	40	H	6:148645188:A:G	70	B	14:35872926:A:G
11	B	2:124346351:G:A^3^	41	Q	6:160113872:A:G	71	D	15:34979959:C:T^2^
12	H	2:130522107:C:G	42	R; K; S	6:160670282:A:C	72	H	15:96571138:T:C
13	H	2:133645476:A:G	43	B	7:45540264:C:G	73	B	16:56524823:A:G^2^
14	D	2:141818657:T:C	44	B	8:9696613:G:T^3^	74	D	16:83573299:A:G
15	C	2:145305277:A:G	45	D	8:48770702:A:C	75	D	17:17545304:A:G
16	I	2:170010985:T:C^+^	46	B	8:102700230:T:C	76	P	17:48768486:A:G
17	J	2:170053505:T:C^+^	47	D	8:141458391:T:C	77	B	17:72240736:T:TA
18	D	2:172034918:A:G	48	B	9:33919723:A:AT	78	W	17:73089852:T:C
19	K	2:178130037:T:G	49	D	9:80397403:G:T^1^	79	D	17:79832479:A:G
20	B	2:179472292:A:T	50	T	9:115986409:T:G	80	D	18:10192951:T:C
21	B	2:186970324:T:TTC​TC	51	B	10:23705239:A:G^1^	81	D	18:51458285:A:G
22	B	3:8013452:A:C	52	N	10:61115114:T:C*	82	B	18:56986855:T:C^1^
23	L; M	3:14187449:T:G	53	B	10:101168886:G:GT	83	B	19:21091519:C:T^1^
24	B	3:141770593:A:G	54	B	10:114893956:C:G	84	M; X	19:45867259:T:C
25	D	4:38940833:A:G	55	H	10:133013187:T:C	85	B	20:21000138:T:C^1^
26	C	4:99561695:T:C	56	B	10:135275358:T:G	86	B	21:33360958:T:TTC
27	D	4:110069218:T:G	57	U; V; W; X	11:67352689:A:G	87	P; E	22:19952132:T:C
28	D	4:182396586:T:G	58	D	12:41419434:A:G	88	O; P; Y	22:19955692:T:C
29	N	4:182621264:A:G*	59	C	12:61795352:T:C			
30	D	4:183127664:T:C	60	B	12:116980314:A:G^3^			

None of the listed variants present in Meta-study reached the Bonferroni-corrected threshold for significance (p < 7.34 × 10^−4^; see Supplementary Table S1). Superscripts indicate the absence of the variant in the Meta-study results:^1^ No proxy variant with an association p-value below the Bonferroni-corrected threshold (p < 5.75 × 10^−4^) was present.^2^ At least one proxy variant with an association p-value below the Bonferroni-corrected threshold was present, but they were not in LD (r2 < 0.1) with the missing variant.^3^ At least one proxy variant with an association p-value below the Bonferroni-corrected threshold was present; however, LD with the missing variant could not be calculated as the missing variant was monomorphic in the LD reference panel.^4^ One of 24 significant proxy variants was absent in the LD reference panel; the rest of the proxy variants were not in LD with the missing variant. * Chromosome and position were used for variant matching across datasets, and allelic information was inferred from dbSNP. ^+^ Variants associated with cisplatin ototoxicity from different studies that are in LD. Studies: (A, Peters et al., 2000; B, He et al., 2023; C, Hurkmans et al., 2023; D, Xu et al., 2015; E, Yang et al., 2013; F, Vos H. et al., 2016; G, Drogemoller et al., 2018; H, Meijer et al., 2021; I, Riedemann et al., 2008; J, Choeyprasert et al., 2013; K, Spracklen et al., 2017; L, Caronia et al., 2009; M, Lopes-Aguiar et al., 2017; N, Garcia et al., 2023; O, Ross et al., 2009; P, Pussegoda et al., 2013; Q, Brown et al., 2015; R, Lanvers-Kaminsky et al., 2015; S, Langer et al., 2020; T, Xu et al., 2012; U, Oldenburg et al., 2007; V, Olgun et al., 2016; W, Drögemöller et al., 2017; X, Lui et al., 2018; Y, Talach et al., 2016).

### Pairwise comparison of genome-wide association results between Pt-study and either Meta-study or Male-study

Comparison association p-value cutoffs of 10^−6^, 10^−5^, 10^−4^, and 10^−3^ were used for pairwise comparison of the datasets. In the base dataset, variants with association p-values below these cutoffs were selected from the clumping results. These variants were then tested for reaching a Bonferroni-corrected threshold for significant association in the target dataset. In variants that reach the threshold for significance, directions of effects for variant alleles were compared in the two datasets. In addition, distributions of the loci lead variants from the base dataset were tested in the QQ plot of the target dataset, including relative to 50th, 99th, and 99.9th percentiles in the target dataset. Coloc package, V5.2.3 (Giambartolomei et al., 2014) (https://CRAN.R-project.org/package=coloc), which performs a Bayesian test for colocalization, was used for the analyses within 250 kb of loci lead variants in the base dataset with association p-value <10^−5^. The default input values for prior probabilities (p1 = p2 = 10^−4^ and p12 = 10^−5^) were used. In the results, a high posterior probability H3 (PP.H3) would indicate that there are two independent association signals in the tested locus across the two studies (i.e., there are two separate causal variants across studies in that locus that are not shared), while a high posterior probability H4 (PP.H4) would indicate that the association signals in the locus are colocalized (i.e., there is a single causal variant across the two studies in that locus). The posterior probability threshold of 0.5 was used in our analyses.

### Potential transcriptional impact of the rs7952909 locus

NCBI Genome Data Viewer (https://www.ncbi.nlm.nih.gov/gdv/) with GRCh37. p13 as the genome reference was used to obtain the protein-coding genes located near rs7952909. GTEx portal (https://gtexportal.org/home/) was used to identify eQTL genes for rs7952909.

The locuszoomr package, V.0.3.5 (https://cran.rstudio.com/web/packages/locuszoomr/) was used to create the local Manhattan plots. PLINK2 was used for clumping and LD calculations with the 1000 genomes as the reference panel. Gene coordinates from the reference package EnsDb.Hsapiens.v75, V2.99.0 (https://bioconductor.org/packages/EnsDb.Hsapiens.v75) were used to plot gene tracks.

The LocusFocus webtool, V1.6.0 (Panjwani et al., 2020) (https://locusfocus.research.sickkids.ca) was used to test colocalization of eQTL associations with association results in Male-study within 250 kb of rs7952909 (11 genes listed). The preloaded GTEx (V7) eQTL datasets across 48 tissues were selected as secondary datasets. The European ancestry of the preloaded 1000 genomes was used for LD calculations. The default test distance of 100 kb (in each direction) from rs7952909 was used for colocalization analysis using the Simple Sum method.

## Results

### Associations of genetic risk factors for cisplatin ototoxicity with hearing difficulty in the general population

From the 88 autosomal variants previously reported to be associated with hearing loss in cisplatin-treated cancer cohorts, 69 variants (representing 68 independent loci) were listed in the Meta-study summary statistics (Table 1). None of these variants demonstrated an association p-value below the Bonferroni threshold for significance (p < 7.4 × 10^−4^) in the Meta-study association results (Supplementary Table S1A). From the remaining 19 variants missing in Meta-study, 11 did not have a significant proxy variant within 500 kb that showed an association p-value below the Bonferroni threshold of significance (p < 5.7 × 10^−4^, Supplementary Table S1B). Eight remaining variants had at least one significant proxy variant. Three of the eight respective loci did not show evidence of linkage disequilibrium (LD) between the proxy variant and the missing cisplatin ototoxicity variant (*r* ^2^ < 0.1) in the 1000 genomes reference panel. The LD for four variants with their proxy variants could not be estimated due to the missing variant being monomorphic in the 1000 genomes. For the last missing variant, no LD was observed with 23 of its 24 proxy variants, with the remaining proxy variant missing in the 1000 genomes (Supplementary Table S1B).

### Comparison of genome-wide association results between Pt-study and Meta- or Male-study

When Meta-study was used as the base dataset (Supplementary Figure S2), none of the variants reached the Bonferroni-corrected threshold for significance in Pt-study. The QQ plot distribution of selected variants shows the presence of 1.74% (10^−6^ cutoff), 1.31% (10^−5^ cutoff), 0.78% (10^−4^ cutoff), and 1.23% (10^−3^ cutoff) of selected variants were among the top 1% variants of the Pt-study associations. When Pt-study was used as the base dataset (Supplementary Figure S3), no variants reached the Bonferroni threshold for significance in the Meta-study results when 10^−6^ and 10^−5^ cutoffs were used for variant selection. In our results, only one variant on chromosome 12 (rs9536379) reached the significance threshold in Pt-study when the 10^−4^ selection cutoff was used. Using the 10^−3^ selection cutoff, four variants reached the threshold for significance, with two of them showing matching directions of effects (rs9536379 and rs10062026) and two showing opposite directions of effects (rs3887874 and rs7138357). QQ plot of variant distribution using the selection cutoff of 10^−5^ shows no variants among the top 1% variants of Meta-study. 1.94% and 0.97% of the selected variants were among the top 1% variants of Meta-study when 10^−4^ and 10^−3^ cutoffs were used, respectively.

**FIGURE 1:**
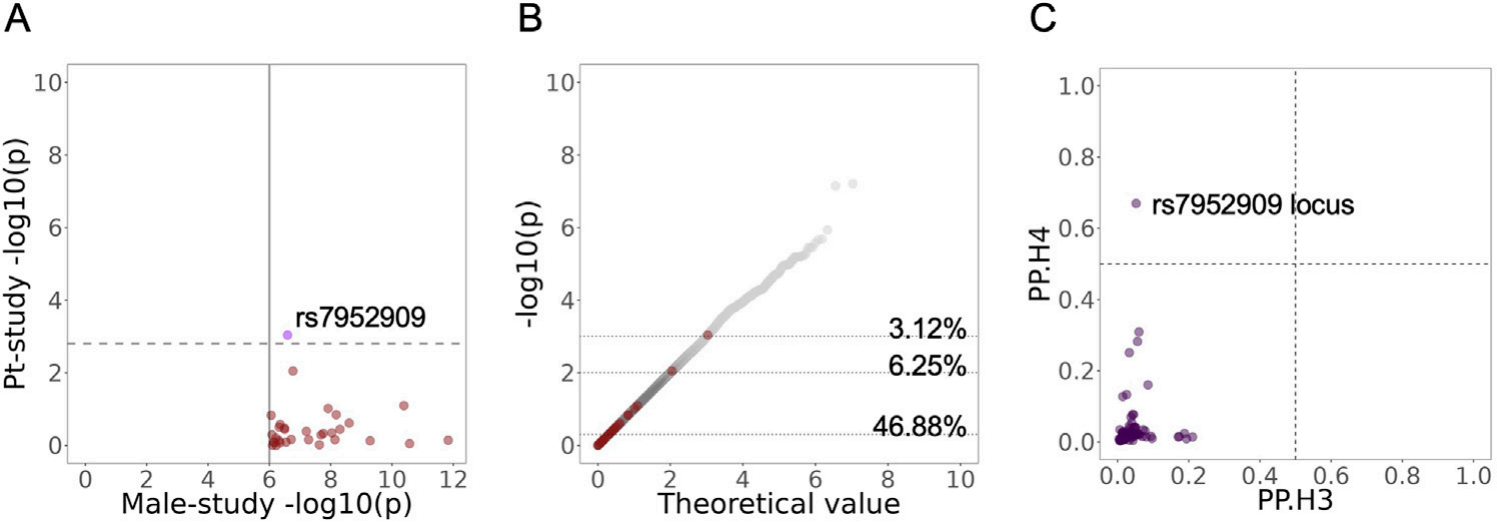
Comparison of the lead associated loci from Male-study in Pt-study. **(A)** Distribution of the loci lead variants with Male-study p-value <10^−6^ in Pt-study. Purple indicates opposite directions of effects in the significant variant. The solid line shows the selection cutoff in Male-study, and the dashed line shows the Bonferroni-corrected threshold in Pt-study. **(B)** Positions of the loci lead variants with an association p-value <10^−6^ (red) from Male-study in the QQ plot of Pt-study (grey). Dotted lines show the 50th, 99th, and 99.9^th^ percentiles in Pt-study, and values next to them show the percentage of loci lead variants from Male-study above the line. For the full panel of pairwise comparison of lead loci variants across studies, see Supplementary Figures S2-S5. **(C)** Colocalization for loci within 250 kb of lead variants with association p-values <10^−5^ in Male-study using the Coloc package. Coloc implements a Bayesian test across the study association statistics, and in its results, the posterior probability H4 (PP.H4) estimates the support for both studies sharing a causal variant in the locus (associations are colocalized), while the posterior probability H3 (PP.H3) value estimates the support for the presence of two separate causal variants in the locus across studies. Dashed lines show posterior probability thresholds of 0.5.

**FIGURE 2:**
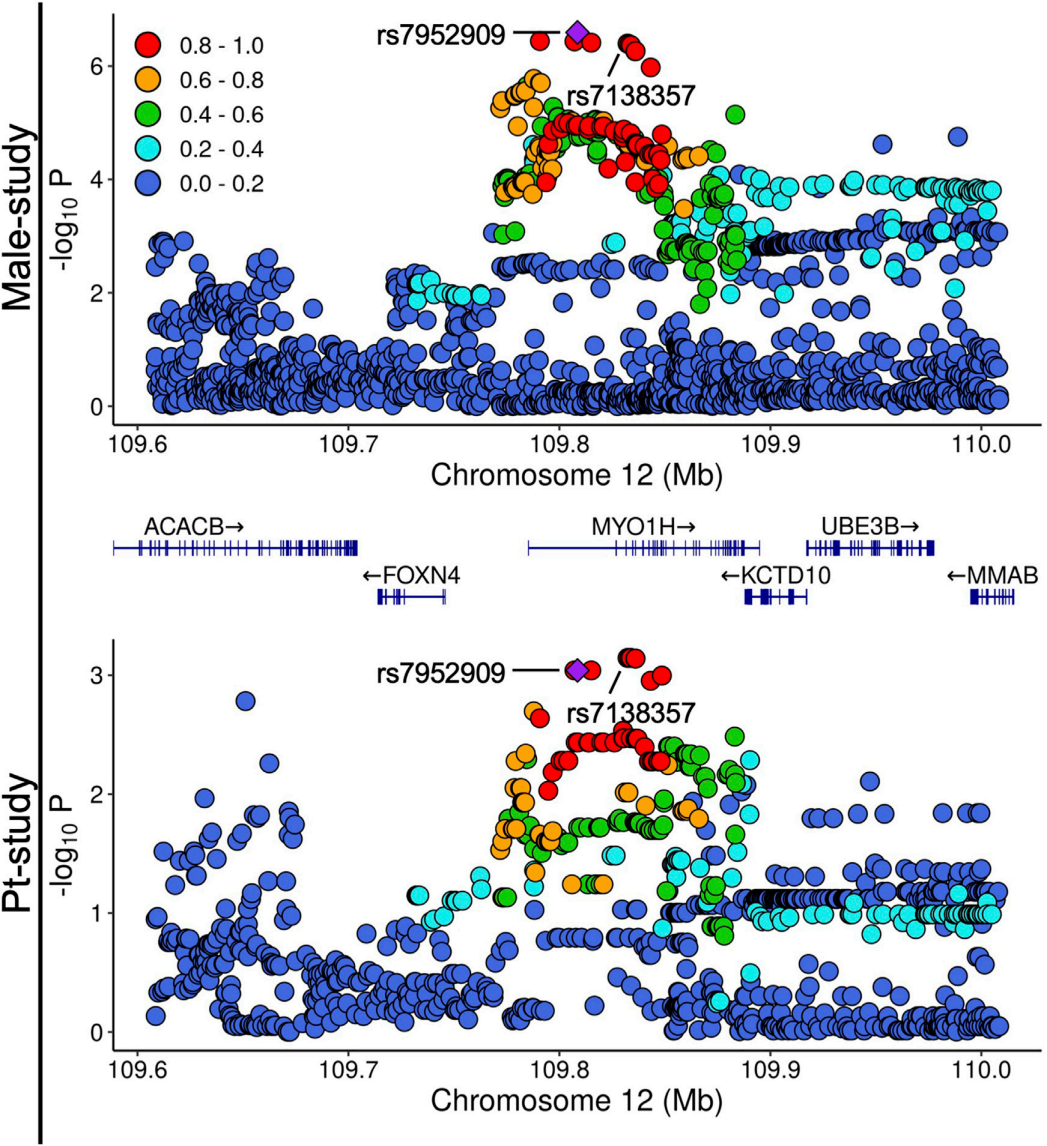
Local Manhattan plots of associations in the rs7952909 locus in Male-study and Pt-study. The color of variants indicates *r* ^2^ of linkage disequilibrium with rs7952909.

In the pairwise comparison of Male-study and Pt-study, when Male-study was used as the base dataset and using the cutoff of 10^−6^ for the selection of variants, rs7952909 reached the Bonferroni threshold for significance in Pt-study, with its effects in opposite directions across the two studies (Figure 1A). Additionally, rs7952909 is also among the top 0.1% associated variants in Pt-study (Figure 1B). When other variant selection cutoffs were used, no other variants reached the thresholds for significance (Supplementary Figure S4). The QQ plot of variant distributions shows that 4.17% (10^−5^ cutoff), 1.87% (10^−4^ cutoff), and 1.52% (10^−3^ cutoff) of the selected variants are among the top 1% of variants in Pt-study. When Pt-study was used as the base dataset (Supplementary Figure S5), no variants reached the Bonferroni threshold for significance when variant selection cutoffs of 10^−5^ and 10^−4^ were used. Meanwhile, two variants reached the threshold for significance when the variant selection cutoff of 10^−3^ was used, with one variant (rs10062026) showing the matching direction of effects and the other variant (rs7138357) showing opposing effects across the two studies. rs7138357 is from the same locus as rs7952909, and they are in high LD (Figure 2). QQ plot of variant distribution using the selection cutoff of 10^−5^ shows no variants among the top 1% variants of Male-study. 0.97% of the selected variants were among the top 1% variants of Meta-study when 10^−4^ and 10^−3^ cutoffs were used.

The colocalization analysis of regions around the top variants indicates colocalization of association signals across Pt-study and Male-study (Figure 1C, posterior probability = 0.67) within the rs7952909 locus. These analyses do not provide evidence for colocalization across association signals in other pairwise comparisons (Supplementary Figure S6).

### Potential transcriptional impacts of rs7952909

The local Manhattan plots in the rs7952909 locus of Male-study and Pt-study are provided in Figure 2. The variant is located within the intronic sequence of *MYO1H*. GTEx portal information indicates that rs7952909 is an eQTL for the expression of 11 genes and an sQTL for the expression of five genes (Supplementary Table S2). The colocalization analysis of local associations with eQTL associations within 100 kb of rs7952909 shows significant associations for nine of the 11 closest genes, including the protein-coding *MYO1H* and the two closest protein-coding genes (*FOXN4*, *KCTD10,* Supplementary Figure S7).

## Discussion

In this study, we set out to determine the extent of overlap among genetic risk factors associated with cisplatin-mediated ototoxicity and hearing impairment in the general population, which would be indicative of the extent of similarity in their genetic architectures. To attain this goal, we created a list of genetic variants that were previously reported to be associated with cisplatin-induced ototoxicity and investigated the association of these variants with hearing impairment in the general population, utilizing the results from a recent meta-analysis (De Angelis et al., 2023). We essentially found that genetic variants associated with hearing loss as a result of cisplatin use are not associated with hearing impairment in the general population, indicating that their genetic risk factors are mainly different. To support this finding, we compared the results from our recent GWAS for hearing loss in a cisplatin-treated cohort (Pt-Study) with genetic association results from the same meta-analysis (Meta-study) or a meta-analysis in the male population (Male-study) in a series of pairwise comparisons. Across these comparisons, only two genetic variants with matching directions of effects reached the significance threshold for replication when relaxed selection cutoffs of 10^−3^ or 10^−4^ were used (this denotes weak associations in the parent dataset). At the more stringent selection cutoff of 10^−5^, we observed evidence for colocalization at the rs7952909 locus across the Male-study and cisplatin-induced ototoxicity study; however, the associations showed opposite directions of effects. These results further support the notion that genetic risk factors for cisplatin-induced ototoxicity and hearing loss in the general population are largely distinct, suggesting different genetic architectures and distinct effects on the underlying cellular and molecular components involved.

### Comparison of genetic risk factors for platinum ototoxicity with those for hearing difficulty in the general population

We evaluated for the first time, to our knowledge, the extent to which shared genetic risk factors exist for cisplatin-induced ototoxicity and hearing impairment in the general population. Sixty-nine variants with reported associations with ototoxicity in platinum cancer cohorts were also listed in the Meta-study results. None reached the threshold of significance for hearing difficulty in the general population. From an additional 19 cisplatin ototoxicity variants that were absent in the Meta-study variants, 14 either lacked a proxy variant that reached the threshold of significance in Meta-study, or their significant proxy variants were not in LD with the missing variants. These observations do not support the presence of a sizable pool of shared genetic risk factors between hearing difficulty in the general population and the previously reported genetic risk factors for cisplatin-induced ototoxicity.

To our knowledge, our Pt-study is the largest cancer survivor cohort evaluating genetic risk factors for the ototoxic effects of cisplatin treatment (Zhang et al., 2022). We, therefore, used the summary statistics from the Pt-study GWAS for pairwise comparisons with the Meta-study results. Considering that Pt-study is a male cohort, we also extended our pairwise comparison to the meta-analysis of hearing impairment in the male population (Male-study) (De Angelis et al., 2023). No variants reached the Bonferroni-corrected threshold of significance when Meta-study was used as the base dataset. Likewise, when Pt-study was used as the base dataset, only four variants from the less stringent selection cutoffs (10^−3^ and 10^−4^) reached the threshold of significance, with only two showing matching directions of effects across the two studies. Interestingly, in the pairwise comparisons of Pt-study and Male-study, with Male-study as the base dataset and the selection cutoff of 10^−6^, rs7952909 reached the threshold for significance in Pt-study with opposite directions of effects across the studies. Another variant from the same locus and in high LD with it reached the threshold of significance when variants from Pt-study were selected for comparison using a selection threshold of 10^−3^. The role of the rs7952909 locus as a shared risk locus with opposite effects was further supported by the colocalization analysis of the association signals in the locus across the two studies. Our observations from pairwise comparisons of association summary statistics might be indicative of the presence of some shared genetic risk factors for hearing difficulty in the general population and hearing loss in platinum-treated patients. However, these shared risk factors seem to constitute a small subset of overall risk factors and are located primarily among the variants that do not exhibit the strongest associations in the parent study.

Due to the significantly higher incidence of ototoxicity in cancer cohorts compared to the general population, an underlying assumption for the identification of the risk factors related to platinum ototoxicity is that most of the associations are driven by cisplatin’s ototoxic effect (and not by risk factors in the general population). The identification of limited shared genetic risk factors with mixed directions of effects supports this assumption and suggests that the genetic risk factors related to platinum ototoxicity likely exert their role in the context of the treatment.

### Potential transcriptional role of rs7952909

The association analyses results from the GTEx Portal provide support that rs7952909 is an eQTL and associated with the expression of 11 genes, and an sQTL associated with splicing in five of these genes. These findings are further supported by colocalization analysis between eQTL associations and the local GWAS summary statistics from Male-study, which indicates significant colocalization of association signals for the expression of nine of the 11 genes in the genomic region surrounding rs7952909.

rs7952909 is an intronic variant for the protein-coding gene *MYO1H.* The two closest protein-coding genes besides *MYO1H* are *FOXN4* and *KCTD10*. Both GTEx results and colocalization analyses support the role of rs7952909 as an eQTL for the expression of these three genes. In the chicken, *MYO1H* is among the differentially expressed genes across the inner-ear hair-cell lineage subtypes (Ellwanger et al., 2018). *FOXN4* encodes a transcription factor that, in mice, plays an essential role during the development of neural tissue, including in the retina (Islam et al., 2013). KCTD10 plays a critical role in brain development in mice, and its expression has been reported in the mouse and human cochlea (Gabashvili et al., 2007; Du et al., 2018; Cheng et al., 2024).

These reports highlight that there are several important candidate genes that could be impacted by genetic variation in rs7952909 and provide the means for rs7952909 to exert its role as a genetic risk factor for hearing loss and platinum ototoxicity. Therefore, it is possible that the diverging effect of rs7952909 across the observed associations is due to a varying role of one or more of the impacted genes in the context of platinum ototoxicity or hearing difficulty in the general population. Alternatively, it is also possible that the diverging effect is due to the independent impact of rs7952909 on separate genes, with varying roles in each condition.

## Limitations

An intrinsic limitation of genome-wide association studies is that they evaluate the direct association of individual variants with the studied trait, thereby limiting their ability to assess complex genetic interactions, such as epistasis. Overfitting is another important limitation in genetic association studies, which may be a contributing factor to the lack of replication of many previously reported variants associated with cisplatin-induced ototoxicity in subsequent studies. The lack of suitable replication cohorts remains another significant challenge for studies evaluating genetic risk factors for chemotherapeutic toxicities. Despite these limitations, we draw our main conclusion by incorporating a large pool of variants and studies, which strengthens our conclusion regarding distinctive pools of genetic risk factors contributing to hearing loss in cisplatin-treated cohorts and hearing impairment in the general population.

A limitation of cancer cohorts for evaluating the genetic aspect of platinum ototoxicity is their relatively small size compared to the large cohorts available to study hearing difficulty in the general population. The reduced power in these studies limits the identification of genetic risk factors and the subsequent evaluation of their role in other conditions. Expansion of current cancer survivor cohorts and the development of larger cohorts can improve future efforts to identify new genetic risk factors and compare their role across conditions.

Cohort-specific characteristics, including age and ancestry, could have confounding effects on the identification of shared genetic risk factors across hearing difficulty and ototoxicity in cancer cohorts. For instance, considering the association of hearing loss with increasing age (Hoffman et al., 2017; Frisina et al., 2021), it is possible that some genetic risk factors play varying roles across different age groups. Therefore, the current study cannot rule out the presence of shared genetic risk factors that were masked due to intrinsic differences in cohorts. The emergence and analysis of population-based and cancer cohorts with higher similarities would be required. Until that time, the existence and extent to which these cohort-specific risk factors contribute to the current evaluation of hearing loss remain unknown.

## Conclusion

Collectively, our results do not support the presence of a sizable pool of common shared genetic risk factors affecting hearing difficulty in the general population and hearing loss in cisplatin-treated cancer cohorts. The absence of a large number of shared genetic risk factors for hearing loss is expected to limit the power and application of predictive genetic models, including polygenic risk score (PRS) models, across cohorts with varying causal environmental risk factors. Further, the absence of a large number of shared risk factors also suggests distinct effects on cellular and molecular components leading to hearing loss, thus supporting research and treatment strategies that allow for the classification of hearing loss based on and in the context of the causal environmental risk factors.

## Data Availability

The summary statistics for Meta-study, Male-study and Pt-study are available from their respective publications (Zhang et al., 2022; De Angelis et al., 2023). The genotype data from the 1000 genomes is a public resource available for download at https://www.cog-genomics.org/plink/2.0/resources. The code for matching variants across genetic association results and clumping is available on GitHub (https://github.com/mshahbazi1/HearingLoss).

## Abbreviations

LD, linkage disequilibrium; eQTLs, expression quantitative trait loci; GTEx, Genotype-Tissue Expression Project; GWAS, genome-wide association study; Male-study, the meta-analysis of genome-wide associations for hearing difficulty in the male population; Meta-study, the meta-analysis of genome-wide associations for hearing difficulty in the general population; Pt-study, the Platinum Study; rsID, reference SNP-cluster ID; SNP, single nucleotide polymorphism; sQTLs, splicing quantitative trait loci.
