# Supplementary material for: Comparison of associations suggests mainly distinct pools of genetic risk factors contribute to cisplatin-induced hearing loss and hearing difficulty in the general population
Source: Front Pharmacol. 2025 Aug 26;16:1577072. doi: 10.3389/fphar.2025.1577072 (PMC12417478; doi:10.3389/fphar.2025.1577072)
Supplement: Supplementary file 2 [file Presentation1.pdf]

Supplemental Figures

Comparison of associations suggests mainly distinct pools of genetic risk factors contribute to cisplatin-induced hearing loss and hearing difficulty in the general population

Mohammad Shahbazi[1], Heather E. Wheeler[2], Xindi Zhang[1], Robert D. Frisina[3], Lois B. Travis[4], M. Eileen Dolan [1*]

[1]Department of Medicine, University of Chicago, Chicago, IL, USA
[2]Department of Biology, Loyola University Chicago, Chicago, IL, USA
[3]Departments of Medical Engineering and Communication Sciences and Disorders, Global Center for Hearing and Speech Research, University of South Florida, Tampa, FL, USA
[4]Department of Medical Oncology, Indiana University, Indianapolis, IN, USA

*** Correspondence:**
M. Eileen Dolan
900 E 57th St., KCBD 7100, Chicago, IL 60637, USA
edolan@bsd.uchicago.edu

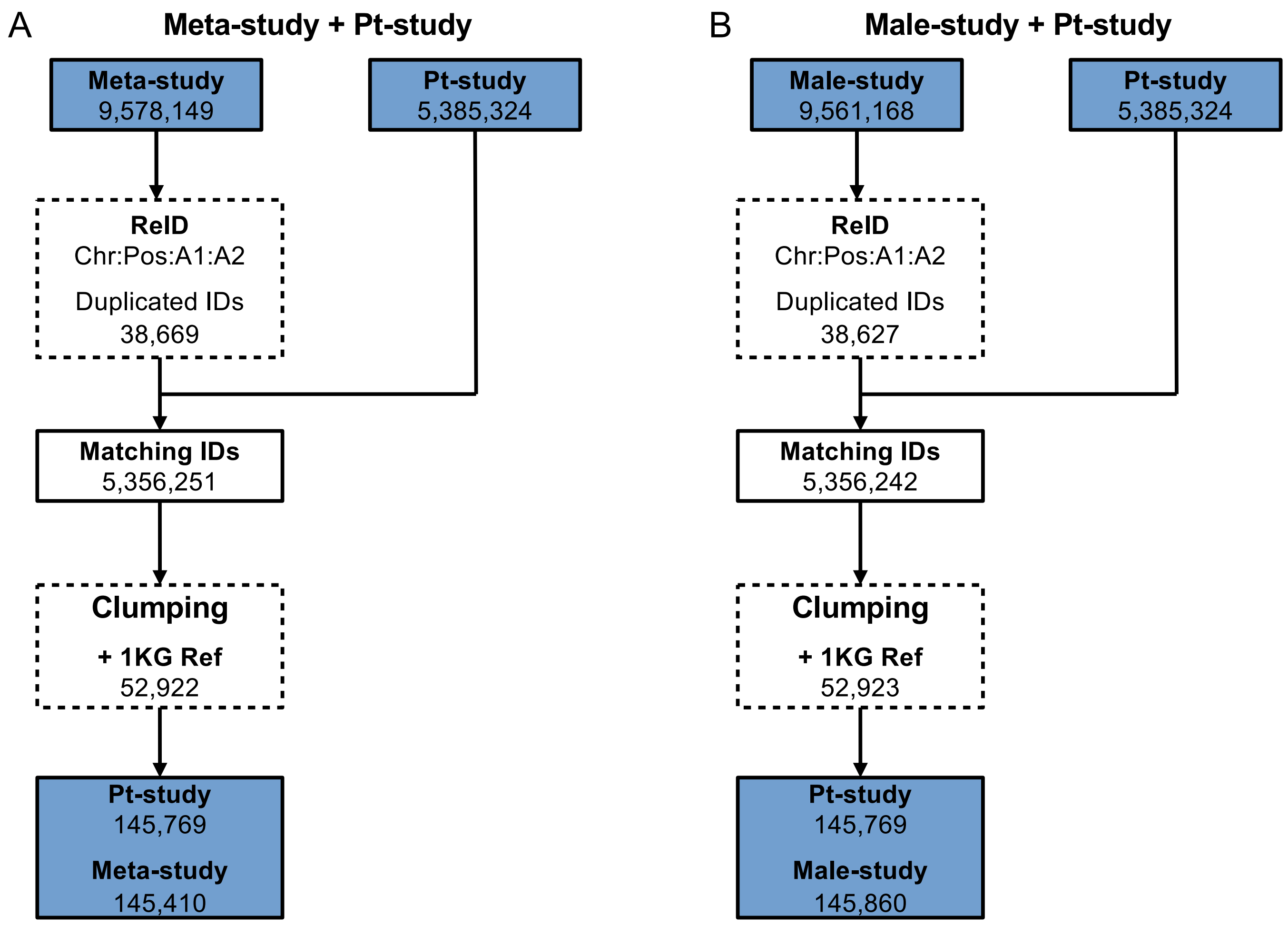

Supplemental Figure 1. Summary of pairwise harmonization and clumping of summary statistics. Dashed boxes indicate filtration steps and the number of variants excluded. 1KG Ref: the 1000 genomes reference panel.

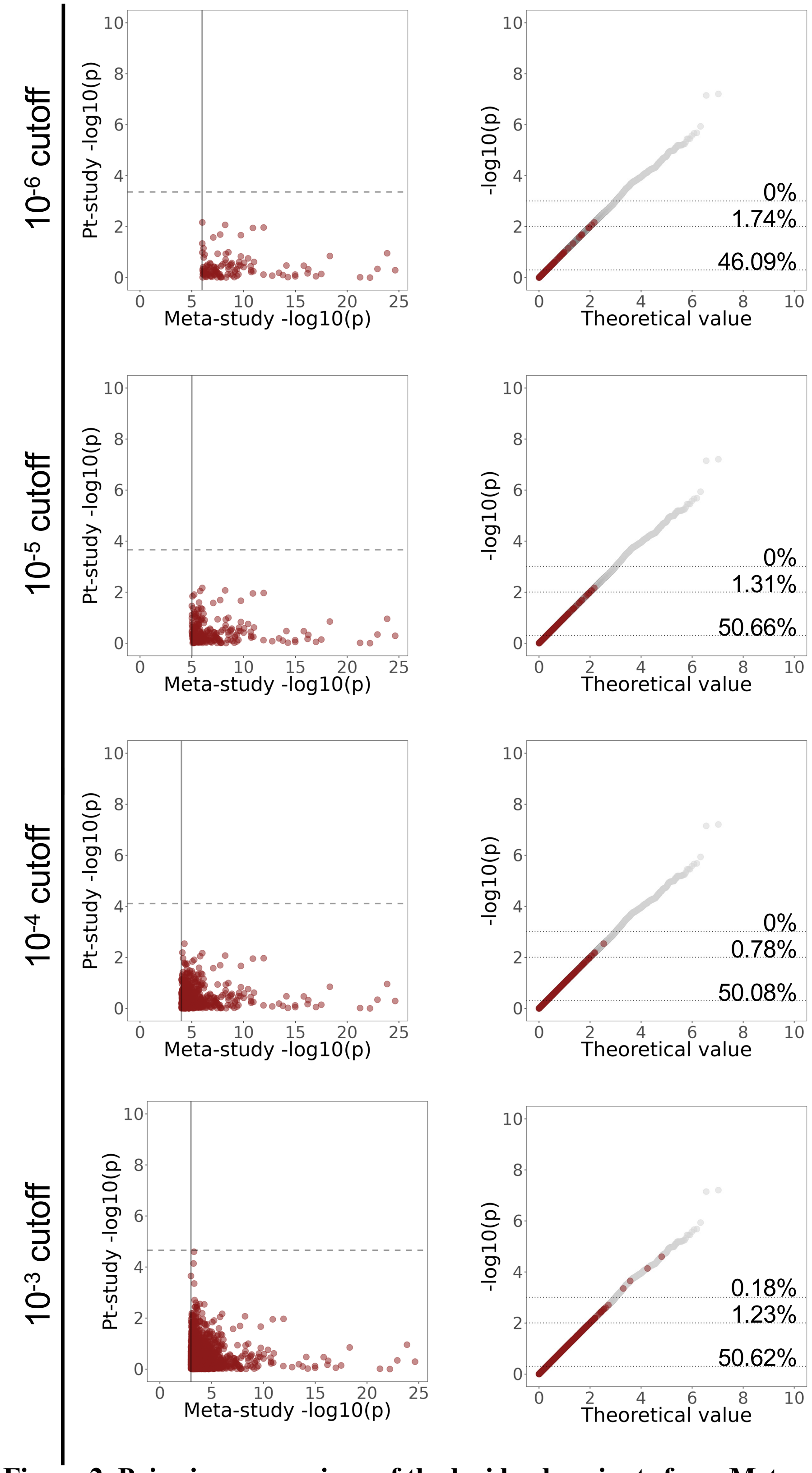

Supplemental Figure 2. Pairwise comparison of the loci lead variants from Meta-study in Pt-study. The left panel shows the distribution of the association p-values for loci lead variants with p-values below the comparison cutoffs in Meta-study. The solid lines show the comparison cutoffs in Meta-study, and the dashed lines show the Bonferroni-corrected thresholds in Pt-study. Blue indicates a match in the directions of effects, while purple indicates opposing directions of effects across the datasets for the significant variants. The right panel shows the position of the selected loci lead variants from Meta-study (red) in the QQ plots of Pt-study (gray). The dotted lines show the 50th, 99th, and 99.9^{th} percentiles in the Pt-study results, and the values next to the lines show percentages of the selected lead variants from Meta-study above them.

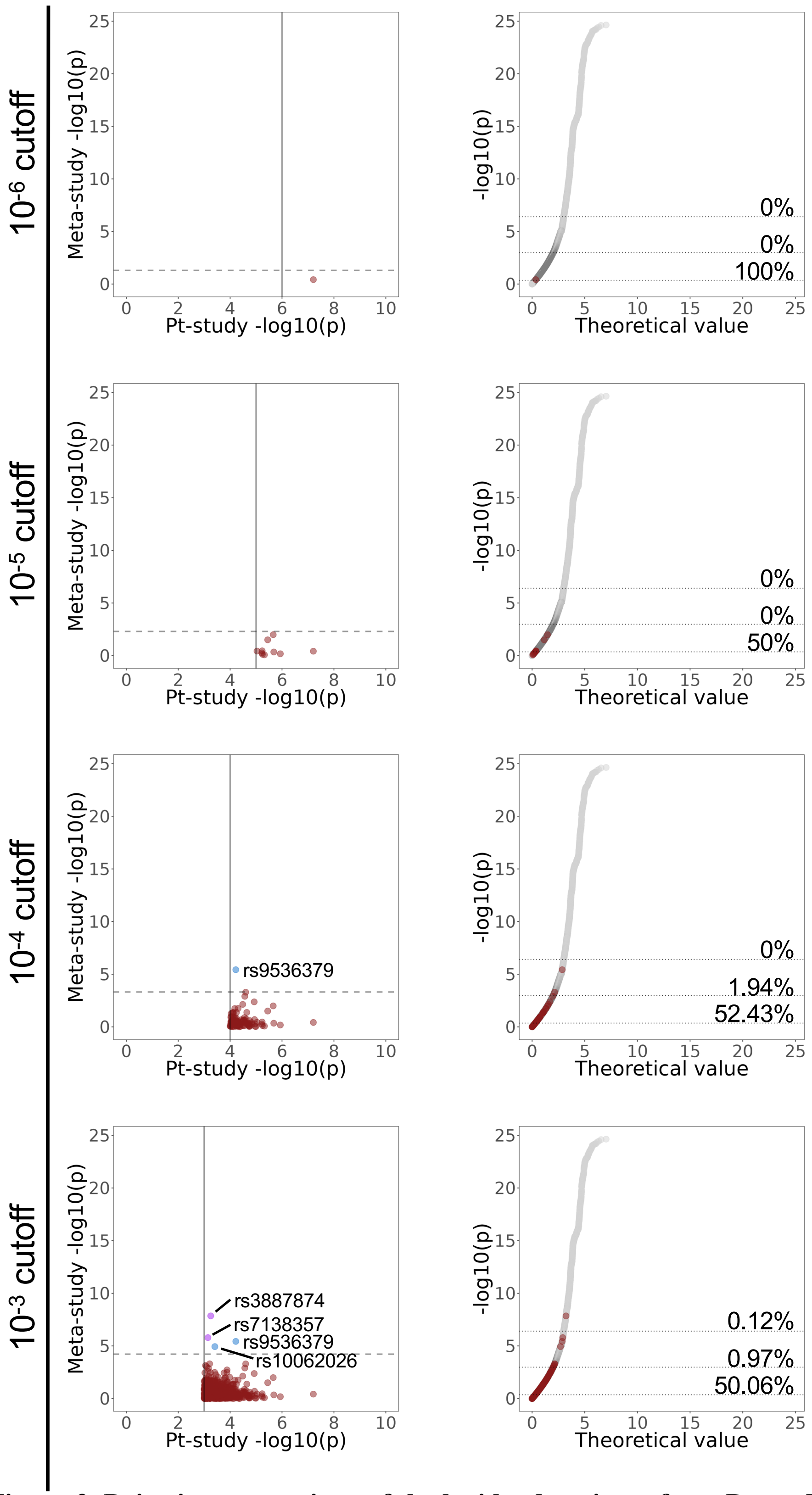

Supplemental Figure 3. Pairwise comparison of the loci lead variants from Pt-study in Meta-study. The left panel shows the distribution of the association p-values for loci lead variants with p-values below the comparison cutoffs in Pt-study. The solid lines show the comparison cutoffs in Pt-study, and the dashed lines show the Bonferroni-corrected thresholds in Meta-study. Blue indicates a match in the directions of effects, while purple indicates opposing directions of effects across the datasets for the significant variants. The right panel shows the position of the selected loci lead variants from Pt-study (red) in the QQ plots of Meta-study (gray). The dotted lines show the 50th, 99th, and 99.9th percentiles in the Meta-study results, and the values next to the lines show percentages of the selected lead variants from Pt-study above them.

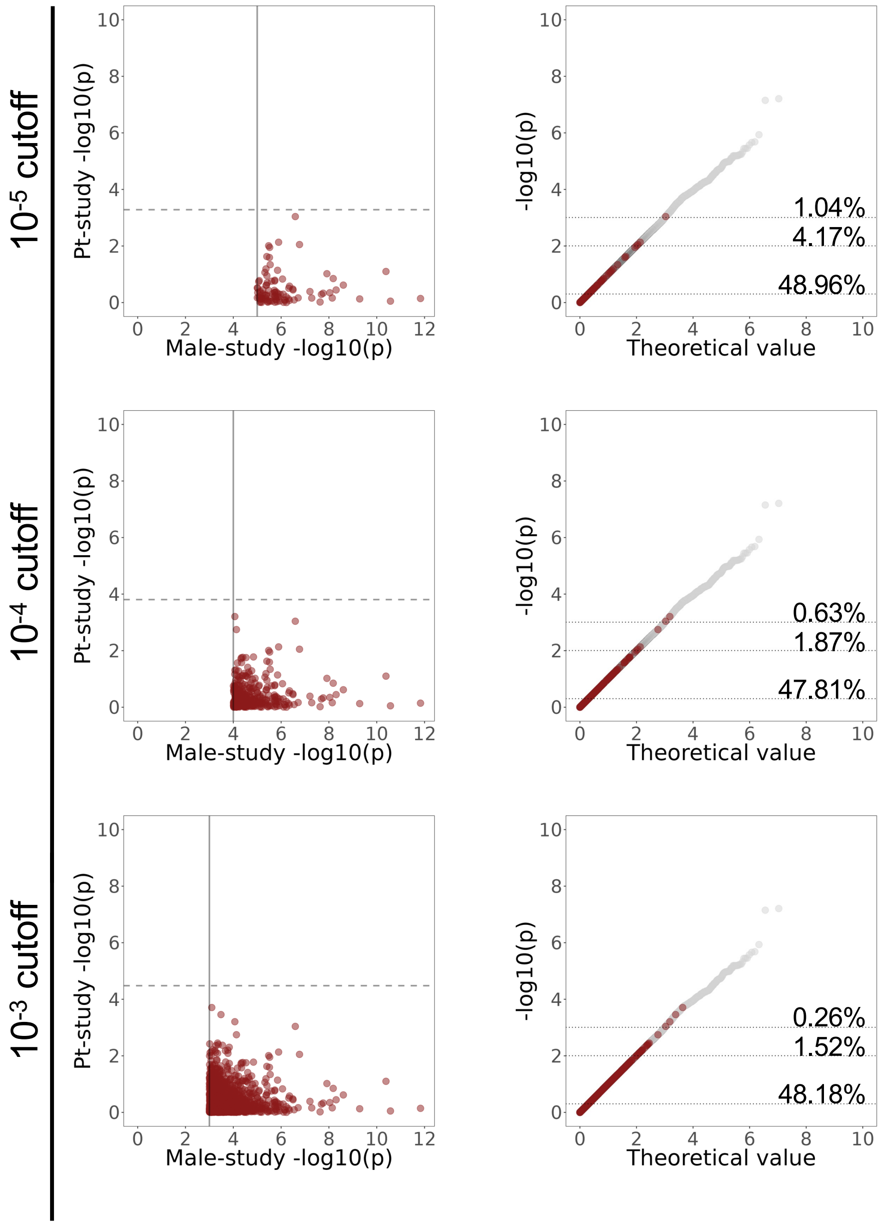

Supplemental Figure 4. Pairwise comparison of the loci lead variants from Male-study in Pt-study. The left panel shows the distribution of the association p-values for loci lead variants with p-values below the comparison cutoffs in Male-study. The solid lines show the comparison cutoffs in Male-study, and the dashed lines show the Bonferroni-corrected thresholds in Pt-study. Blue indicates a match in the directions of effects, while purple indicates opposing directions of effects across the datasets for the significant variants. The right panel shows the position of the selected loci lead variants from Male-study (red) in the QQ plots of Pt-study (gray). The dotted lines show the 50th, 99th, and 99.9th percentiles in the Pt-study results, and the values next to the lines show percentages of the selected lead variants from Male-study above them.

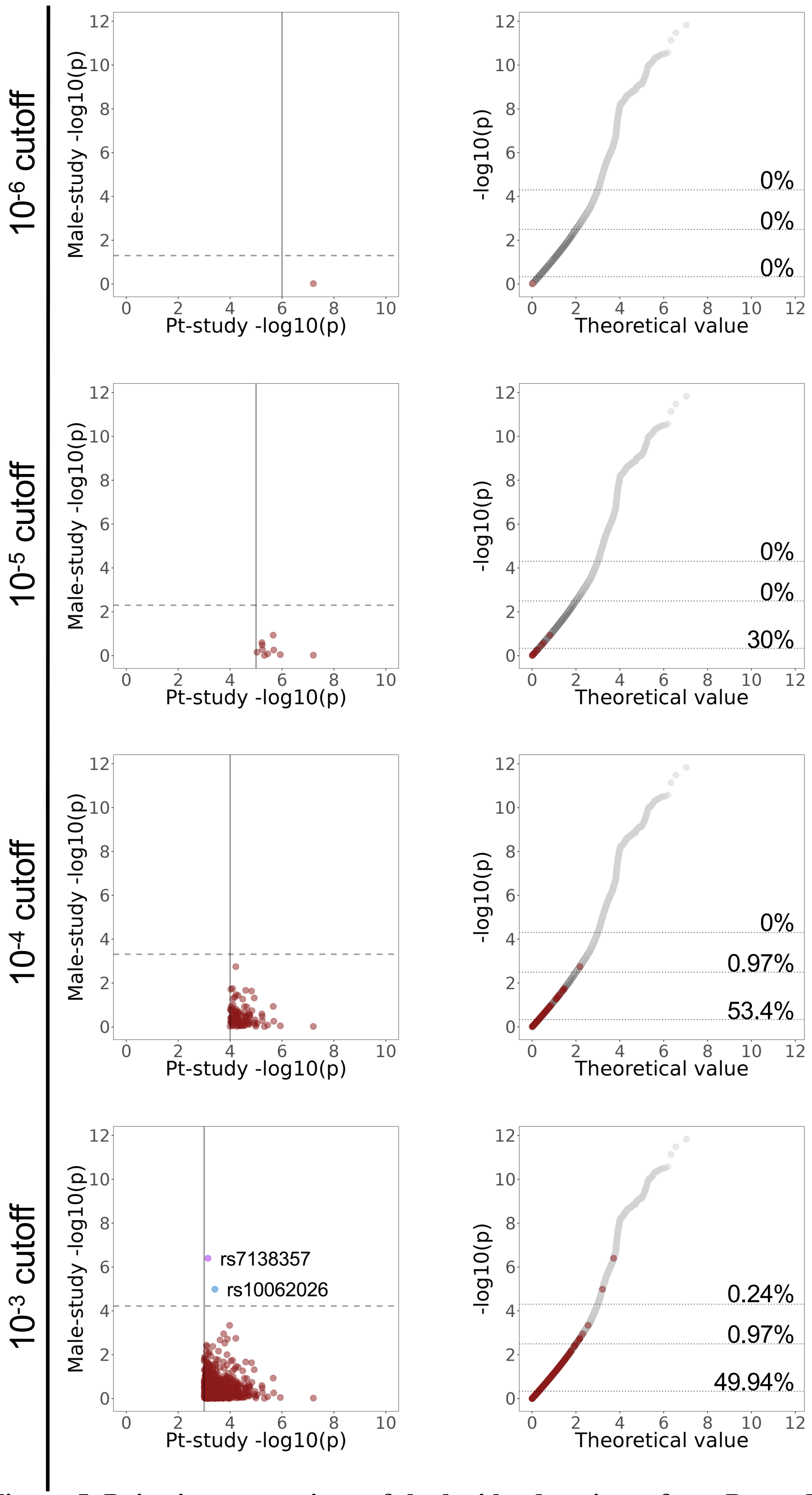

Supplemental Figure 5. Pairwise comparison of the loci lead variants from Pt-study in Male-study. The left panel shows the distribution of the association p-values for loci lead variants with p-values below the comparison cutoffs in Pt-study. The solid lines show the comparison cutoffs in Pt-study, and the dashed lines show the Bonferroni corrected thresholds in Male-study. Blue indicates a match in the directions of effects, while purple indicates opposing directions of effects across the datasets for the significant variants. The right panel shows the position of the selected loci lead variants from Pt-study (red) in the QQ plots of Male-study (gray). The dotted lines show the 50th, 99th, and 99.9th percentiles in the Male-study results, and the values next to the lines show percentages of the selected lead variants from Pt-study above them.

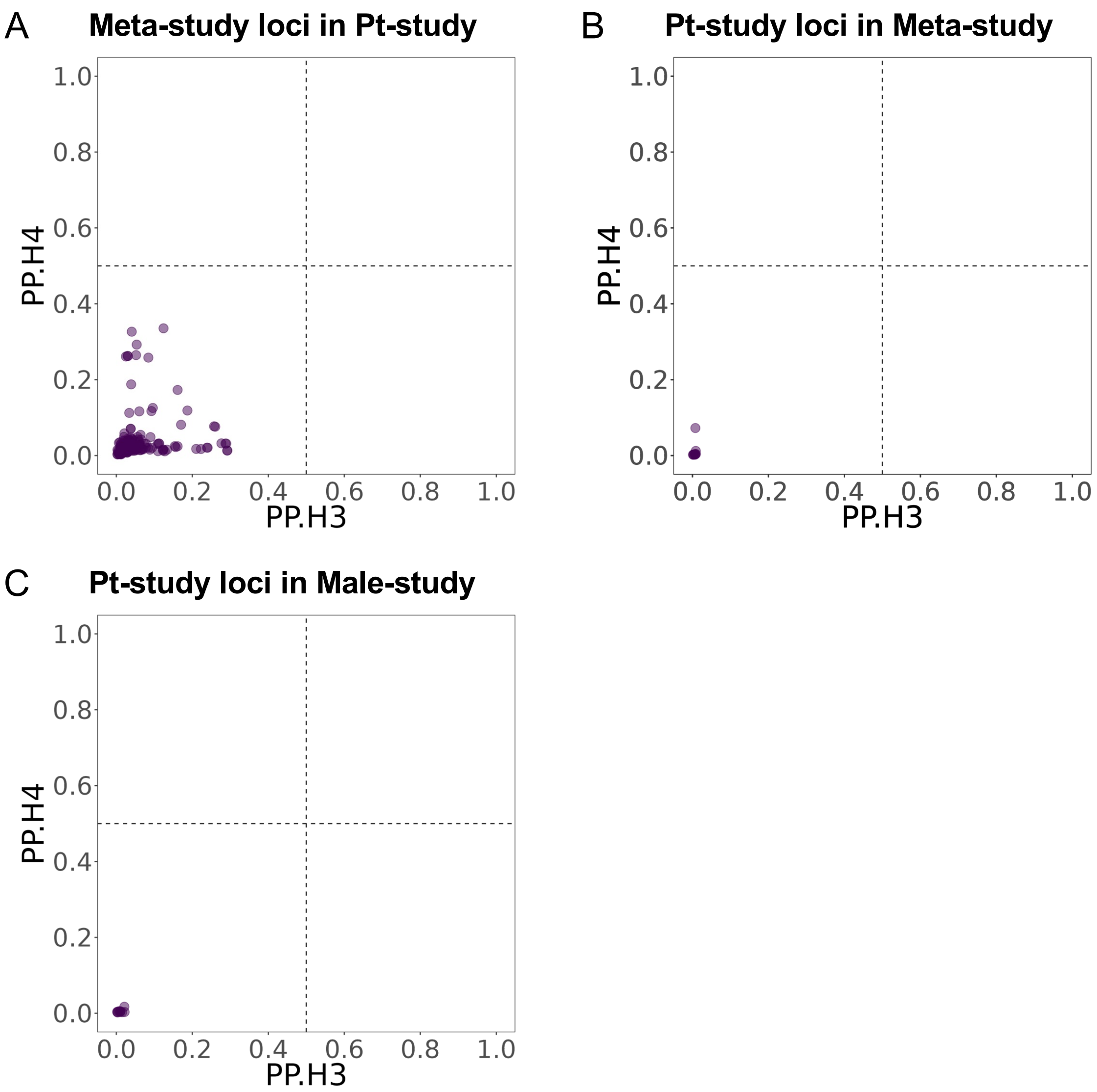

Supplemental Figure 6. Pairwise colocalization for loci within 250 kb of lead variants with an association p-value < 10^{-5} in the base dataset. (A) Meta-study as the base dataset and Pt-study as the target dataset **(B)** Pt-study as the base dataset and Meta-study as the target dataset. **(C)** Pt-study as the base dataset and Male-study as the target dataset. Dashed lines show posterior probability thresholds of 0.5. PP.H4 is the posterior probability indicating the colocalization of association signals, and PP.H3 is the posterior probability indicating the presence of two independent association signals in the locus.

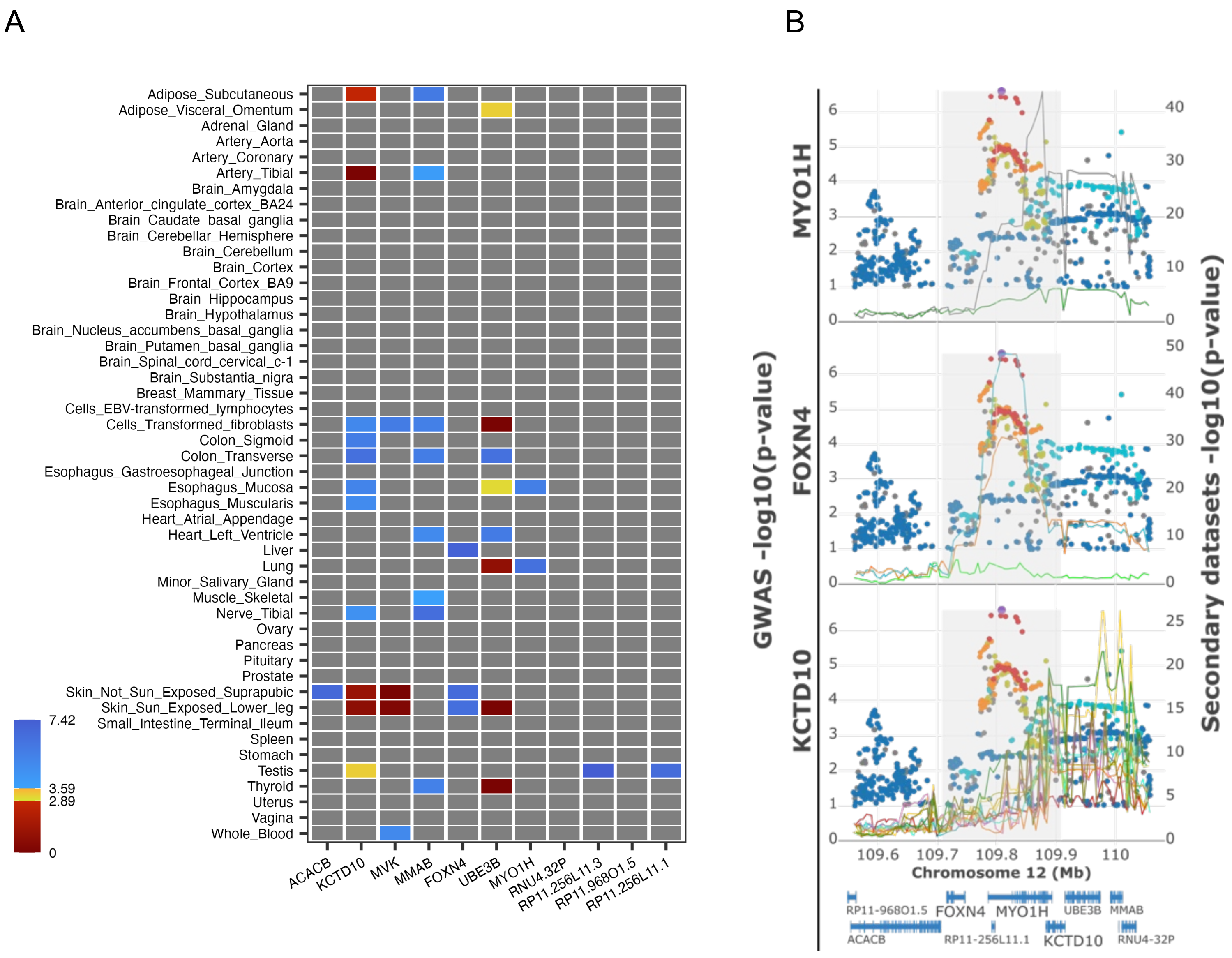

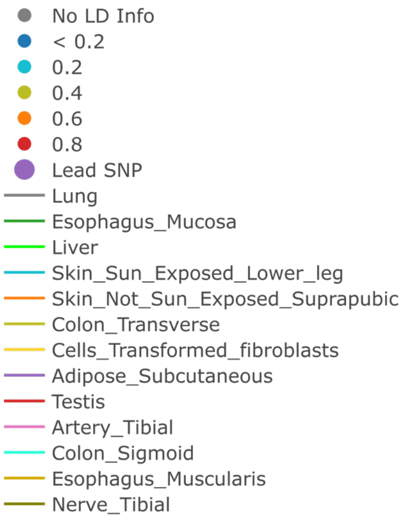

Supplemental Figure 7. LocusFocus colocalization of associations in Male-study and eQTL associations at the rs7952909 locus. (A) Heatmap of the p-values for the Simple Sum colocalization analyses. Gray boxes indicate removal by LocusFocus during the first-step filtration. 2.89 and 3.59 represent the 0.05 and 0.01 cutoffs, respectively, after the Bonferroni adjustment for multiple testing. **(B)** Local Manhattan plots for the three nearest protein-coding genes, with trendlines following variant associations with gene expression in the tissues that showed significant colocalization. The gray box indicates the region used for the colocalization test.
